# Treatment gap, help-seeking, stigma and magnitude of alcohol use disorder in rural Ethiopia

**DOI:** 10.1186/s13011-019-0192-7

**Published:** 2019-01-18

**Authors:** Selamawit Zewdu, Charlotte Hanlon, Abebaw Fekadu, Girmay Medhin, Solomon Teferra

**Affiliations:** 1 Department of Psychiatry, Addis Ababa University, College of Health Sciences, School of Medicine Addis Ababa, Addis Ababa, Ethiopia; 2 Debre Markos University, College of Health Sciences, Debre Markos, Ethiopia; 3 Centre for Innovative Drug Development and Therapeutic Trials for Africa (CDT-Africa), College of Health Sciences, Addis Ababa University, Addis Ababa, Ethiopia; 4 Global Health & Infection Department, Brighton and Sussex Medical School, Brighton, UK; 5 Centre for Global Mental Health, Institute of Psychiatry, Psychology and Neuroscience, King’s College London, London, UK; 6 King’s College London, Institute of Psychiatry, Psychology and Neuroscience, Centre for Affective Disorders, London, UK; 7 Addis Ababa University, Aklilu Lemma Institute of Pathobiology, Addis Ababa, Ethiopia; 8 Harvard T.H. Chan School of Public Health, Boston, MA USA

**Keywords:** Alcohol use disorder, Community-survey, Help-seeking, Barriers, Stigma, Prevalence ratio, PRIME-Ethiopia

## Abstract

**Background:**

Although alcohol use disorders contribute a high proportion of population disease burden, the treatment gap is large, especially in low- and middle-income countries. To narrow this gap, contextually relevant evidence is needed to inform service development in low- and middle-income country settings. The aim of this study was to assess the magnitude of the treatment gap for alcohol use disorder, help-seeking behavior, stigma and barriers to care among people with alcohol use disorder in rural Ethiopia.

**Methods:**

A cross-sectional, house-to-house survey was conducted in Sodo district, south Ethiopia. A sample of 1500 adults was selected using simple random sampling from a census of households and screened for alcohol use disorder using the alcohol use disorders identification tool (AUDIT). Help-seeking, barriers to care and internalized stigma were investigated among people with moderately severe alcohol use disorder (AUDIT score ≥ 16). Poisson regression with robust variance was used to examine factors associated with alcohol use disorder.

**Results:**

The prevalence of alcohol use disorder (AUDIT ≥8) in the past 12 months was 13.9% (25.8% in men and 2.4% in women, *p*-value < 0.001). People with alcohol use disorder had increased disability (adjusted prevalence ratio (aPR) 1.03, 95% confidence interval (CI) 1.01, 1.03) and higher depressive symptom scores (aPR 1.02, 95% CI 1.01, 1.04). The treatment gap was very wide, about 87.0% (only 13% sought help) of participants with an AUDIT score ≥ 16 had never sought help for their alcohol problems and 70.0% reported high internalized stigma. Major barriers to seeking help were wanted to handle the problem on their own, believing that it would get better by itself and being unsure about where to go.

**Conclusions:**

Although alcohol use disorders are common problems in Ethiopian community, the unmet need for treatment is substantial. An integrated care approach has the potential to address this need, but stigma and low awareness may be major barriers to help-seeking. Interventions to reduce stigma and enhance community awareness are recommended.

## Background

Alcohol is not an ordinary substance of abuse [1]: alcohol is integrated into the culture of many societies, is widely used and causes significant medical, psychological, and social harm on a global scale [1, 2]. Alcohol consumption is the third highest risk factor for global disease burden, associated with 5.1% of disability adjusted life years (DALYs) [3]. One in 20 deaths globally (5.9% of all deaths and injuries) were attributed to alcohol drinking in the year 2012 [2]. In countries with lower economic status, the disease burden is higher per liter of pure alcohol consumed; up to 70.0% of alcohol-related deaths occur in low and middle income countries (LMICs) [2, 4].

Alcohol is associated with more than 200 avoidable health conditions, of which more than 30 occur solely because of alcohol consumption [2]. When consumed at a high level (10–20 g per day), alcohol is associated with increased risk of cancers, liver cirrhosis, injuries and heart disease [2, 4, 5]. Alcohol also increases the risk of contracting communicable diseases, such as human immunodeficiency virus (HIV) and tuberculosis [2, 4]. Within alcohol-specific conditions, alcohol use disorders (AUDs) are the most significant and account for half of alcohol related harm [5].

Alcohol use disorder (AUD) is defined as a problematic pattern of alcohol use within a 12-month period, which is manifested as an increase in drinking for a long time, unable to control drinking, development of tolerance, experience of withdrawal symptoms and continuation of use even when alcohol drinking causes problems in social life and harm to physical or psychological health [6].

About 5% of adults globally [7] and an estimated 3.0% of the African population meet criteria for AUDs [2]. A recent national survey in Ethiopia reported that 12.4% of people age 15 or more had heavy episodic drinking (six or more drink in single occasion in the past 30 days) [8]. In a community based survey in rural Ethiopia, more than one in five people (21.0%) were hazardous drinkers [9].

Despite the major burden caused by AUD, AUDs remain neglected, with most people untreated, even in high-income countries [10–16]. The treatment gap for AUDs was estimated to be about 78% [13] globally. However, in LMICs, this figure is estimated to be about 95% [16, 17]. People with AUDs often make contact with primary health care (PHC) for the physical and mental health consequences of their condition, but these disorders are rarely identified by health professionals in primary health care [10, 18]. Once an AUD is detected, people may not be treated or take up the service due to a number of reasons, including the accessibility and availability of services.

Even when the service is available, people may fail to seek help. Evidence suggests that significantly greater barriers exist to receipt of mental health care in comparison with physical health care. Some of the factors that increase the likelihood of treatment avoidance, delays to care, and discontinuation of service use include: lack of knowledge about the features and treatability of the disorder; ignorance about how to access assessment and treatment; prejudice against people who have the disorder, and expectations of discrimination against people who have a diagnosis. Substance abuse is consistently associated with high rates of public stigma and internalized stigma and institutional discrimination that may discourage people from getting health care. [17, 19–21]

To narrow the wide treatment gap and improve help seeking behavior among people with AUD, it is important to explore the reasons for not seeking treatment [10–13]. However, there is dearth of evidence from Ethiopia and other LMICs.

Therefore, the aim of this study was (i) to assess the magnitude of AUD, comorbid problems and associated factors, and (ii) to determine the treatment gap, help-seeking behavior and barriers to accessing care among people with AUDs to inform development of a future task-shared service for AUDs integrated into primary care.

## Methods

This study was carried out as part of the formative phase of the Programme for Improving Mental Health CarE (PRIME) [22]. PRIME is a consortium of research institutions and Ministries of Health in five countries in Asia and Africa (Ethiopia, India, Nepal, South Africa and Uganda), with partners in the UK and the World Health Organization (WHO).

### Study design and setting

A community-based, cross-sectional survey was conducted from March to April 2014, in Sodo district, Gurage Zone, Southern Nations, Nationalities and Peoples’ Region (SNNPR), which is located about 100 km south of Addis Ababa. Sodo is a rural district with different climatic zones and is the second most populous district in the SNNP Region. The district had 161,952 inhabitants (79,356 men and 82,596 women) during the study period, who were living in 58 sub-districts (*kebeles*) [19]. The majority (97.0%) of the population is Orthodox Christian, from the Gurage ethnic group (85.3%) and engaged mostly in subsistence farming [23]. There are eight health centers, with eight to 24 health professionals per facility. The average number of people served by each health center is around 20,000. In addition to the health centres, each of the 58 sub-districts has one health post staffed by community health workers called health extension workers [23]. At the initiation of PRIME, there was no mental health or alcohol treatment service in the district [24]; people needed to travel to Addis Ababa to access alcohol treatment services. However, an integrated mental healthcare program has started with the support of the PRIME project [24].

### Participants and sample size

Adults aged 18 years and above, who had lived for at least six months in the district and were willing to give consent were included in the study. The sample size was determined by assuming a 10% prevalence [25] of AUDs, with a design effect of 1.5, anticipated non-response rate of 15%, and a power of 0.8 to detect 5 to 25% changes in treatment coverage for common mental disorders (CMD) as a result of interventions of PRIME. This yielded a final sample size of 1500.

One thousand five hundred households were selected with the simple random sampling method from the district. We employed health extension workers coordinated by the district office to carry out a complete census by visiting every house and listing all members of the households in each sub-district (*kebele*). We then computerized this list and used it as a sampling frame. After the households were selected, one participant was selected per household using lottery method.

### Data collection and variables

Instruments were piloted and pre-tested. After the pilot we were able to identify some concepts that needed re-wording and changes needed to the response categories. It also helped us to decide on the number of data collectors that we need to finalize the data collection without compromising on the length of the questionnaire and to decide on the number of questionnaires to be completed by one data collector per day. The measures were administered in Amharic by 34 trained lay interviewers, supervised by four trained and experienced supervisors, three PhD students and two research assistants. Self-reported age, sex, residence, duration of residence, marital status, educational status, income, perceived relative wealth and occupation. The following measures were used:

### Alcohol use disorder

Alcohol consumption in the past 12 months was assessed using Alcohol use disorder identification tool (AUDIT), a 10-item screening tool developed by the World Health Organization (WHO) [26]. The AUDIT assesses alcohol consumption in terms of standard drinks, drinking behaviors, and alcohol-related problems.

Each item was rated on a five-point scale, with the total score ranging from zero to 40. A score of eight or more on the AUDIT indicates the presence of a probable AUD. A score 8–15 indicates a medium level of alcohol problem, score 16 or more indicates high level of alcohol problems. A score of 20 or more on the AUDIT requires further diagnostic assessment for possible alcohol dependence [27].

The AUDIT has been validated across genders and in a wide range of racial/ethnic groups and LMIC settings. [26, 28, 29]. Originally AUDIT was developed for the PHC setting; however, it has been found to be reliable and valid as a screening tool in the general population [30] and widely used [31–33].

PRIME made country-specific adaptations for AUDIT as recommended by the World Health Organization. The traditional beverages in Ethiopia were converted to equivalent alcohol units previously [34]. Homemade alcoholic drinks in the setting include *‘tella’* (alcohol content 2–4%), *tej* (alcohol content 7–11%) and distilled liquor ‘*areqi*’ (alcohol content 45%). These beverages are consumed at home during social and religious ceremonies and holidays, and in small traditional outlets called ‘*mesheta bet*’ or ‘*tella bet*’ or *‘areqi bet’* especially during market days and/or while farming [9]. A chart illustrating the approximate number of standard drinks in different alcohol beverages, adapted for the Ethiopian context [34], was included for reference and used during data collection. The AUDIT was found to have a high Internal consistency in this study (Cronbach’s α = 0.84).

### Co-morbid conditions

#### Depression

Participants were screened using the Patient Health Questionnaire (PHQ-9) for depression [31]. The reference time for the scale is the last two weeks. Each item on the PHQ-9 is scored 0 (not at all), 1 (several days), 2 (more than half the days) and 3 (nearly every day), with the total score ranging from 0 to 27. The PHQ-9 has been validated in the Ethiopian primary care setting [32, 33].

#### Suicidality

Suicidal behavior (ideation, plan and attempt) was measured using the WHO Composite International Diagnostic Interview (CIDI) [34]. This instrument was adapted for assessing the 12-month prevalence of suicidal behavior. The CIDI has been used in previous Ethiopian studies [35, 36].

#### Disability

Disability was assessed using the World Health Organization Disability Assessment Schedule (WHODAS) version 2.0, with 12 items [37]. The instrument assesses functioning in the previous 30 days. The WHODAS covers the functional domains of understanding and communicating, getting around, self-care, getting along with people, life activities, and participation in society. Each item was scored from zero (none) to four (extreme or cannot do). Using the polytomous scoring method, the sum of all items was divided by 48 and multiplied by 100 to yield the total WHODAS score. A higher score indicates greater severity of disability.

### Psychosocial factors

#### Stressful Life events

The List of Threatening Experiences (LTE) questionnaire was used to measure twelve commonly encountered stressful or major life events [35]. The time period for consideration was the last six months. This instrument has been adapted and used previously in the Ethiopian setting [9, 25].

#### Social support

Social support was assessed using the three-item Oslo Social Support (OSS) questionnaire [36]. The OSS has three items related to the number of people that they can count on, people showing concern, and how easily they can get help from people in the neighborhood. The total score ranges between three and 14. Scores from three to eight are considered as poor support, scores from nine to 11 are considered to be average support, and a score between 12 and 14 is considered to be strong social support. The OSS has been used in the same setting and showed good utility [9, 25, 37].

### Help seeking behavior

For participants with an AUDIT score ≥ 16, a single question asked, “Did you seek any help for these problems?” Follow-up questions then asked about the time, type, source and usefulness of the help sought.

### Treatment gap

The difference between true prevalence and treated prevalence is the treatment gap. In this study, treatment gap was operationalized as the percentage of people with alcohol use disorder (defined as scoring 16 or more on the AUDIT) who did not get treatment or any help from any source.

### Barriers to care

For participants with an AUDIT score ≥ 16, barriers to care were assessed using the Barriers to Accessing Care Evaluation (BACE) scale [38]. The original BACE has response categories from 0 (not at all) to 3 (a lot) for 31 possible barriers to care. PRIME adapted and modified BACE as “Yes” and “No” question for 21 potential barriers for seeking professional care in the setting.

The BACE covers a range of issues that have a potential role to stop, delay or discourage an individual from getting, or continuing with professional care for a mental health problem. Potential barriers include individual perception and knowledge and attitudes about the care, stigma, infrastructure, social support and previous experiences. This instrument was used previously in the Ethiopian setting [37].

### Internalized stigma

For participants with an AUDIT score ≥ 16, internalized stigma was assessed using the Internalized Stigma of Mental Illness Inventory (ISMI) [39] which was adapted for the PRIME community survey to be applicable to people with AUD. The ISMI has response categories from 1 (strongly disagree) to 4 (strongly agree) for 11 items from the original 29 item scale. The overall mean was calculated and used as a cut-off value to categorize respondents into “higher” and “lower” internalized stigma. The ISMI was previously used and found to be valid and reliable in Ethiopian context [40].

### Statistical analysis

Socio-demographic characteristics were summarized descriptively using the appropriate measure of central tendency or proportions for the entire study sample and for the sub-sample who scored ≥16 on AUDIT.

Odds ratios (OR) from a cross-sectional study might overestimate the effect when the outcome is common (> 5% prevalence), and so prevalence ratios were calculated, as recommended [41–44]. To obtain prevalence ratios [42, 45–48], Poisson regression with robust variance was conducted.

Multiple Poisson regression was used to adjust for possible confounding of pre-specified variables. After establishing a full model, the adjusted effects of suicide, depressive symptom score on PHQ-9 and total disability score on WHODAS were obtained by including each in the full model separately. Crude and adjusted prevalence ratios with corresponding 95% confidence intervals and *p*-values were reported.

Help-seeking behavior, barriers to seeking professional help and internalized stigma in participants with AUDIT score ≥ 16 were investigated. The association between help seeking among this participants and socio-demographics characteristics, depression, suicide, disability and internalized stigma was analyzed using separate bivariate Poisson regression.

Multivariable analysis was not carried out in this sub-sample because of the small number of people with an AUDIT score ≥ 16. Analyses were performed using STATA version 13 (STATA for Windows) [49]. Sampling weights were used to calculate the prevalence of AUD and while fitting Poisson regression models.

## Results

### Socio-demographic characteristics and psychosocial factors

Of 1500 individuals approached for interview, 1485 (99.0%) completed the interview. The mean age of study participants was 39.3 years (standard deviation (SD) 15.3). Just over half (54.3%) of the participants were female, 74.3% were currently married, 89.6% resided rurally, 93.3% were Gurage by ethnicity, 70.5% did not attend formal education and 92.3% were followers of Ethiopian Orthodox Christianity (Table 1).

**Table 1:** Socio- demographics characteristics of participants in Sodo, Gurage Zone, South Ethiopia

Characteristics		Total N (%) *n* = 1485	Alcohol use disorders	
			AUDIT ≥8 Yes	AUDIT ≥16 Yes
			N (%) n = 203	N (%) *n* = 57
Age (in years)	< 25	207 (13.9)	22 (10.6)	2 (3.5)
	25–34	427 (28.6)	34 (8.02)	8 (14.0)
	35–44	369 (24.8)	55 (27.1)	16 (28.1)
	45–54	205 (13.8)	33 (16.3)	10 (17.5)
	≥55	280 (18.9)	59 (29.1)	21 (36.8)
Sex	Female	806 (54.3)	21 (10.3)	2 (3.5)
	Male	679 (45.7)	182 (89.7)	55 (96.5)
Marital status	Married and living together	1104 (74.3)	168 (82.8)	48 (84.2)
	Never Married	178 (12.0)	23 (11.3)	6 (10.5)
	Widowed	136 (9.2)	5 (2.5)	2 (3.5)
	Divorced	37 (2.5)	4 (2.0)	0
	Married & not living together	30 (2.0)	3 (1.5)	1 (1.8)
Residence	Rural	1331 (89.6)	188 (92.6)	50 (87.7)
	Urban	154 (10.4)	15 (7.4)	7 (12.3)
Education level	Not literate	719 (48.4)	54 (26.6)	14 (24.6)
	Able to read and write	325 (21.9)	73 (36.0)	19 (33.3)
	Primary	321 (21.6)	60 (29.6)	21 (36.8)
	Secondary	92 (6.2)	15 (73.9)	3 (5.3)
	College/university	24 (1.6)	1 (0.4)	0
Occupation type	Housewife	337 (22.8)	4 (2.0)	2 (3.5)
	Farmer	734 (49.7)	154 (76.2)	44 (77.2)
	Merchant	145 (10.4)	14 (6.9)	2 (3.5)
	Day laborer	130 (8.8)	23 (11.4)	2 (3.5)
	Students	46 (3.1)	0	5 (8.8)
	Civil servant	35 (2.4)	3 (1.5)	0
	Others ^a^	41 (2.8)	4 (2)	2 (3.5)
Religion	Orthodox	1371 (92.3)	201 (99.0)	57 (100.0)
	Protestant	71 (4.8)	1 (0.5)	0
	Muslim	41 (2.8)	1 (0.5)	0
	Others ^b^	2 (0.1)	0	0
Ethnicity	Gurage	1384 (93.3)	189 (93.1)	51 (89.4)
	Oromo	66 (4.4)	12 (5.9)	5 (8.8)
	Amhara	23 (1.5)	2 (1.0)	1 (1.8)
	Others^c^	11 (0.7)	0	0
Perceived relative wealth	Lower	658 (44.7)	84 (41.4)	25 (43.9)
	Average/the same	714 (48.5)	104 (51.2)	30 (52.6)
	Better off	99 (6.7)	15 (7.4)	2 (3.5)

percentages un-weighted

a- includes priest and other unspecified works; b- includes Wolayta, Tigrayan; c -includes Catholic, no religion,

From those who had AUD, 20.7% (*n* = 48), 50.8% (*n* = 100) & 28.4% (*n* = 55) had poor, average and strong social support respectively. The most frequently reported stressful events during previous six months were financial crisis, being upset, death of relative and serious illness.

### Prevalence of alcohol use disorder and comorbid conditions

The total AUDIT score ranged from zero to 35, with a median AUDIT score of one [interquartile range (IQR) 0–4]. The weighted prevalence of AUD in the last12 months (score of ≥8 on the AUDIT) was 13.9% (*n* = 203) (95% CI 10.9 to 17.3%), with a significant gender difference (25.8%, *n* = 182 among males and 2.4% *n* = 21 among females; *p*-value < 0.001). Based on level of drinking, 9.9% (*n* = 146) had probable hazardous alcohol use (AUDIT score 8–15), 2.2% (*n* = 30) had probable harmful alcohol use (AUDIT 16–19) and 1.8% (*n* = 27) had probable dependence (AUDIT ≥20). In the previous 12 months, 23.3% (*n* = 355) of the study participants had binge drinking (drinking at least six alcoholic drinks on a single occasion). Almost all of those who scored at least 16 on AUDIT were men (96.5%) (55 of 57) (Table 1). Total WHODAS score ranged from zero to 83.3 and median score was 6.2 (IQR = 0–18.7). Over a quarter (38%, *n* = 80) of people with AUD scored at least 5 on PHQ-9 and the 12-month prevalence of suicidal ideation was 10.5% (*n* = 23) in those with AUD (≥8 on AUDIT). In the 23 study participants who had AUD and suicidal ideation, sixteen (75.0%) had attempted suicide.

### Treatment gap, help-seeking behavior and barriers to seeking care

**Fig. 1:**
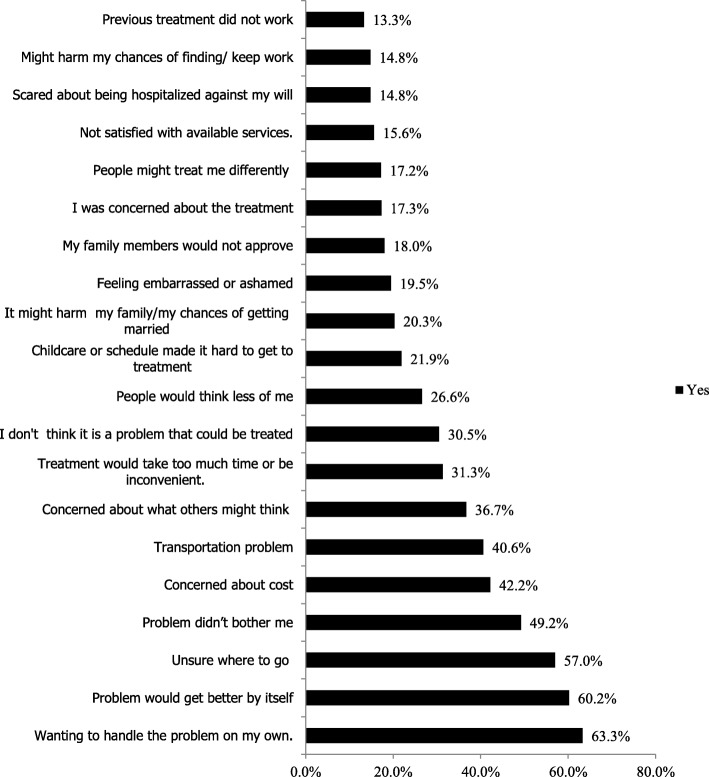
Barriers for seeking professional help among adults with AUDIT score ≥ 16 in Sodo, Gurage Zone, South Ethiopia

The treatment gap was wide, only 13.0% (6/57) had sought help for their alcohol problem at least once in their lifetime from a health center (*n* = 2), from a hospital (n = 2) or in a religious setting (*n* = 2). About 47 (81%) out of 57 participants reported at least one barrier to seek professional help. The median number of barriers was 4 [IQR 3–6]. The main barriers to seeking professional help for alcohol problem were wanting to handle the problem by themselves (63.3%), thinking that the problem would get better by itself (60.2%), not being bothered by the problem (57.0%), feeling unsure where to go (49.2%) and being concerned about cost of professional help (42.2%) (Fig. 1).

Among 57 study participants who scored at least 16 on AUDIT, 70.3% (n = reported high internalized stigma due to their drinking: 77.1% (*n* = 37) agreed that they were disappointed in themselves, 56.3% (*n* = 27) felt embarrassment and 45.8% (*n* = 22) agreed that they were thinking that they could not achieve much in life because of these problems (Table 2).

**Table 2:** Internal stigma beliefs among adults with AUDIT score ≥ 16 in Sodo, Gurage Zone, South Ethiopia

Internalized stigma beliefs, *n* = 48	Agree or strongly agree N (%)
I am disappointed in myself due to these problems	37 (77.1)
I am embarrassed or ashamed of these problems	27 (56.3)
Others think that I cannot achieve much in life because of these problems	22 (45.8)
People ignore me or take me less seriously just because of these problems	21 (43.8)
I feel out of place in the world because of these problems	19 (39.6)
These problems have spoiled my life	18 (37.5)
People often patronize me, or treat me like a child, just because of these problems	17 (35.4)
I cannot contribute anything to society because of these problems	15 (31.3)
Nobody would be interested in getting close to me because of these problems	13 (27.1)
Because of these problems, I need others to make most decisions for me	13 (27.1)
People discriminate against me because of these problems	11 (22.9)

### Factors associated with alcohol use disorder

In the final multivariable analysis, older age, male gender and occupation (being a farmer, trader or daily laborer) were associated independently with AUDIT score ≥ 8 (Table 3). Alcohol used disorder were more prevalent in men (aPR = 7.7), farmers (aPR = 3.9), traders (aPR = 6.0) and daily laborers (aPR = 6.3) when compared to housewives. Oneyear increase in age is associated with 1% increase in the prevalence of AUDs (aPR = 1.01; 95% CI: 1.00, 1.02).

**Table 3:** Factors associated with alcohol use disorder (AUDIT ≥8) in Sodo, Gurage Zone, South Ethiopia

		Having AUD %	Crude PR (95% CI)	Adjusted PR (95% CI)
Socio-demographic characteristics (Model A)				
Age (in years)	Mean = 39.4 Yrs. (SD 15.3)		1.02 (1.01, 1.02)*	1.01 (1.00, 1.02)*
Sex	Male	25.8	10.9 (6.7, 17.7)*	7.7 (4.4, 13.1)*
	Female	2.4	Reference	Reference
Educational status	Formal education	16.6	Reference	Reference
	No formal education	12.7	0.7 (0.5, 1.0)	1.0 (0.7, 1.4)
Occupation type	Housewives	0.90	Reference	Reference
	Farmer	21.1	24.6 (7.7, 78.6)*	3.9 (1.0, 14.8)*
	Trader	9.10	11.2 (3.1, 39.9)*	6.0 (1.5, 23.9)*
	Daily laborer	17.7	21.0 (6.2, 71.0)^*^	6.39 (1.5, 26.1)*
	Others^a^	5.7	5.6 (1.4, 22.2)*	1.9 (0.4, 9.0)
Perceived relative wealth	Lower	12.3	1.3 (0.9, 2.0)	0.6 (0.3, 1.0)
	Average/the same	15.0	1.2 (0.9, 1.7)	0.8 (0.5, 1.3)
	Better-off	17.7	Reference	Reference
Residence	Rural	14.2	1.3 (0.7, 2.3)	1.4 (0.7, 2.5)
	Urban	10.4	Reference	Reference
Marital status	Currently married	15.4	Reference	Reference
	Others^b^	9.3	0.6 (0.4, 0.8)	0.8 (0.5, 1.3)
Social support	poor	16.1	1.3 (0.9, 2.0)	1.3 (0.9, 1.9)
	Average	14.7	1.2 (0.9,1.7)	1.2 (0.9, 1.6)
	strong	11.6	Reference	Reference
Stressful life events	Mean = 3.8 (SD 1.4)		1.3 (1.2, 1.3)*	1.2 (1.1, 1.3)*
Factors adjusted for model A				
Suicidal ideation	Yes	13.3	1.7 (1.1, 2.6)*	1.5 (1.1, 2.1)*
	No	23.5	Reference	Reference
Total depression symptom score (PHQ-9), median = 2.0, IQR 0 to 5			1.06 (1.03, 1.09)*	1.06 (1.02, 1.09)*
Total disability score (WHODAS), median = 6.5, IQR 0 to 18.6			1.02 (1.00,1.02)*	1.02 (1.00, 1.04)*

AUD-Alcohol use disorder (AUDIT≥8): PHQ-9 - Patient Health Questionnaire version 9; WHODAS _ World Health Organization Disability Assessment Schedule; SD-Standard Deviation; PR – Prevalence Ratio, IQR – interquartile range a-includes single, widowed, divorced; b- includes students, civil servants and others; * significant at *p* value < 0.05; P value 0,027 for age, < 0.001 for total depression symptom score and 0.010 for disability score; Sampling weight used during analysis

Alcohol use disorder was also positively and significantly associated with higher number of stressful life events, higher total depression symptom score, higher disability and suicidality. As the number of stressful events increase by one, the prevalence of AUD increased by 27% (aPR = 1.27; 95%CI: 1.1, 1.3).

Every one increase in depressive symptom score (PHQ-9) and in a total disability score on WHODAS are associated with 3.0 and 2.0% increase in prevalence of AUD respectively. Having suicidal thought also associated with AUD (aPR = 1.5; 95%CI: 1.1, 2.1).

## Discussion

In this population-based study from a rural Ethiopian district, there was evidence of a high prevalence of alcohol use disorders, large treatment gap, low help-seeking behavior and high levels of internalized stigma in people with AUD. The main reported barriers to care included: wanting to handle the problem by themselves, thinking that the problem would get better by itself, not being bothered by the problem, feeling unsure where to go and financial barriers, including being concerned about cost of professional help.

The prevalence of AUDs was higher than studies carried out previously in Butajira which was 3.7% [50] and 2.7% in Addis Ababa Ethiopia [51]. In these previous studies, the sample included younger people (15 or more years) and more than half of the sample in Butajira was Muslim, compared to the current study sample which was predominantly Orthodox Christian. The prevalence was, however lower than that seen in a recent study [9] conducted in the same setting. The current study used a culturally adapted standardized measure, the AUDIT, which has been shown to have high sensitivity and specificity in a range of LMICs, but in the previous study the Fast Alcohol Screening Test (FAST) was used which has high sensitivity but lower specificity than AUDIT [52].

In line with previous studies in LMICs, increasing age, male gender, stressful events, suicidality, disability and severity of depressive symptoms [32, 33], were significantly and positively associated with AUDs. As the study was cross-sectional, it is not possible to elucidate the direction of association: alcohol problems might cause stressful events or stressful events might lead a person to drink as a coping mechanism. Similarly, depressive symptoms, disability and suicidal ideation could be a cause or effect of AUD.

Stigma due to AUDs and its relation with help-seeking has not been studied well in LIMCs [21]. In this study, high internalized stigma was reported by most of participants with an AUDIT score of 16 or more. Stigma attached to being called an ‘alcoholic’ may lead people to avoid seeking help and contribute to low utilization of treatment services, since it confirms their membership to the stigmatized group. In line with the PRIME studies from India and Uganda [31, 32], the main stigmatizing beliefs endorsed by participants were being disappointed by oneself, feeling of embarrassment or shame, others thinking that they cannot achieve much in life because of alcohol problems, ignored by people or taken less seriously just because of these problems and feeling out of place in the world because of these problems.

### Implication of the study

Evidence on interventions targeted to reduce AUD in LMCs is scarce [10]. The findings from this study have implications for the development and implementation of alcohol interventions in this rural Ethiopian setting. Interventions targeted at changing public awareness about alcohol use disorders, the treatment and where it is offered need to be developed and implemented in parallel with strengthening the integration of alcohol treatment services into routine health care service.

Integration will make the series accessible and may reduce barriers associated with cost, which was mentioned as a barrier to seeking help. Interventions for the reduction of both internalized and experienced stigma are also likely to be important to promote the uptake of services.

Evidence-based population level interventions, including the introduction of regulations for availability of alcohol (including locally made beverages) in the market and alcohol advertising may be helpful to reduce the high prevalence of the disorder [2, 10].

This study must be interpreted with consideration of the following limitations: Although there was an attempt to standardize the alcohol content of the local drinks [34], the alcohol content of locally made drinks may vary. Self-report questionnaires administered in an interview-format may be prone to social desirability and recall bias which could potentially influence prevalence estimates. The AUDIT is a screening tool and a clinical diagnosis would have been preferable but was not feasible for a population level study. The reverse causality between AUDs and depressive symptoms, disability and some of the stressful life events cannot be ruled out because of the cross sectional study design. Help seeking behavior was determined among a very small number of people with high levels of AUD; this may affect the generalizability of the result.

## Conclusions

In conclusion, although alcohol use disorder is a common problem, it is left untreated. The unmet need for treatment is substantial. Integrating alcohol treatment services into PHC settings is important to address this need, since people with alcohol problem are not coming for treatment of the AUD but tend to have frequent contact with PHC settings. However, stigma and low awareness may be major barriers to help-seeking. Interventions targeted at changing public awareness about alcohol use disorders, the treatment, where it is offered and stigma reduction are recommended to promote the uptake of alcohol treatment services. Screening at community level also useful to detect cases at early stage and prevent further consequence of the problem. Regulation of locally-produced alcoholic beverages is also important. Good quality prospective studies are also recommended to assess the link between depression, suicidality and disability with AUDs in local setting. Qualitative studies are also important to explore barriers to care, and the effect of stigma on AUDs and help seeking.

## Abbreviations

aPR: Adjusted prevalence ratio
AUD: Alcohol use disorder
AUDIT: Alcohol use disorders identification tool
BACE: Barriers to Accessing Care Evaluation
CI: Confidence interval
CIDI: Composite International Diagnostic Interview
CMD: Common mental disorder
DALYs: Disability adjusted life years
FAST: Fast Alcohol Screening Test
HIV: Human immunodeficiency virus
IQR: Interquartile range
ISMI: Internalized Stigma of Mental Illness Inventory
LMICs: Low and middle income countries
LTE: List of Threatening Experiences
OR: Odds ratio
OSS: Oslo Social Support
PHC: Primary health care
PHQ-9: Patient Health Questionnaire
PRIME: Programme for Improving Mental Health CarE
SD: Standard deviation
SNNPR: Southern Nations, Nationalities and Peoples’ Region
SPSS: Statistical Packages for Social Sciences
WHO: World Health Organization
WHODAS: World Health Organization Disability Assessment Schedule
